# Changing patterns of cancer burden among elderly across Indian states: Evidence from the global burden of disease study 1990–2019

**DOI:** 10.1002/agm2.12264

**Published:** 2023-08-21

**Authors:** Chandan Kumar Swain, Sourav Padhee, Umakanta Sahoo, Himanshu Sekhar Rout, Prafulla Kumar Swain

**Affiliations:** ^1^ Department of Analytical & Applied Economics Utkal University Bhubaneswar Odisha India; ^2^ Department of Statistics Utkal University Bhubaneswar Odisha India; ^3^ Department of Statistics Sambalpur University Sambalpur Odisha India; ^4^ RUSA Centre of Excellence in Public Policy and Governance Utkal University Bhubaneswar Odisha India

**Keywords:** burden of cancer, DALY, elders, Indian states, prevalence rate

## Abstract

**Objective:**

To investigate the trends and patterns of the cancer burden among the elderly in different regions of India at a subnational level.

**Methods:**

Data were extracted from the Global Burden of Disease (GBD) Studies India Compare 2019. Prevalence rate, disability‐adjusted life years (DALY), and annual percentage change techniques were used to analyze data.

**Results:**

The three age groups with the highest prevalence of cancer were those aged 60–64 years, 65–69 years, and 70–74 years. In 2019, The prevalence of cancer among the elderly ranged from 7048.815 in Karnataka to 5743.040 in Jharkhand. Kerala has the most significant annual percentage change in the cancer prevalence rate of 0.291 between 1990 and 2019. The highest DALY rate was observed among individuals aged 80–84 years in 2019. That year, the DALY rate among the elderly was 8112.283 in India. The top five cancers with higher DALY rates among the elderly in India in 2019 were tracheal, bronchus, and lung cancer (908.473), colon and rectum cancer (752.961), stomach cancer (707.464), breast cancer (597.881), and lip and oral cavity cancer (557.637).

**Conclusion:**

Elderly individuals demonstrated a higher vulnerable to cancer compared to other age groups. There is a need for state‐specific government intervention to minimize the risk of cancer among the elderly due to the heterogeneity in the burden of cancer across Indian states.

## Graphical abstract

To investigate the trends and patterns of the cancer burden among the elderly in different regions of India at a sub‐national level. This study compiled data from the Global Burden of Disease (GBD) Studies India Compare 2019. Prevalence rate, disability‐adjusted life years (DALY), and annual percentage change techniques were used to analyze data.

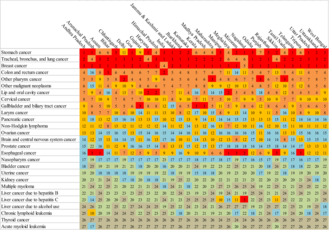

## 1 INTRODUCTION

A significant epidemiological transition has been seen where the burden of noncommunicable diseases (NCDs) is now spreading to developing countries that were once limited to developed countries only.^1^ NCDs account for 71% of all deaths in the world, and in India, it contributes about 69% of the total mortality. Among these NCDs deaths, cancer has emerged as a leading cause of death in India, responsible for 9% of NCDs‐related death.^2^ Moreover, the cancer share in the burden disease has risen from 3.4% in 1998 to 5% in 2016, as measured by disability‐adjusted life years (DALY).^3^ Additionally, other studies found a twofold increase in the proportion of cancer in the burden of diseases between 1990 and 2016. Although the age‐standardized incidence rate for cancer cases remained unchanged during this period, there was a 2.6‐fold change in the burden of the disease in terms of DALY rates across different states in India.^4^

In 2011, the incidence of cancer in India reached a total of 1,193,426 cases, with a higher occurrence among females (603,560) compared to males (589,866). It has been forecasted that there will be an estimated 1,869,983 new cancer cases, with 934,268 cases among males and 935,715 cases among females by 2026.^5^ Cancer cases can be categorized into two types: infectious and noninfectious. Infectious cancers are stomach cancer, cervical cancer, and liver cancer, while noninfectious cancers are lung cancer and breast cancer. As the country develops, there is a shift in the burden of cancer from infectious to noninfectious causes. Noninfectious cancers are primarily influenced by changes in age distribution and lifestyle‐related factors.^6^, ^7^ Consequently, the incidence rate of breast cancer has experienced a significant increase of 40.7% between 1990 and 2016, while the incidence rates of stomach cancer, lip and oral cavity cancer, cervical cancer, and leukemia have shown a declining trend in India.^4^ In addition, late marriage and dietary patterns have been identified as potential contributing factors to the rise in breast cancer.^8^, ^9^, ^10^, ^11^

Geographical variation in the burden of cancer was observed across different regions of India.^4^, ^12^ The prevalence of specific cancers, such as breast, colorectal, and prostate cancer, tends to be higher in industrialized societies.^13^, ^14^ The average age at which breast and ovarian cancer typically developed was around 45–50 years in India, which was 10 years younger compared to developed nations where the onset age is typically over 60 years.^15^ The incidence rate for ovarian cancer did not substantially increase for individuals aged 30–64 years between 1976 and 2005.^16^ Additionally, cervical cancer is the most common cancer among women aged between 30 and 69, accounting for 17% of all cancer‐related deaths.^13^, ^14^ However, the incidence rate of cervical cancer declined in India over the period.^16^, ^17^ Other risk factors such as smoking and physical inactivity are associated with a higher incidence of cancer.^18^

The rise in the global elderly population was due to improvements in life expectancy over the past few decades.^19^ The elderly had a higher burden of cancer compared to other age groups.^5^, ^18^, ^20^, ^21^, ^22^ Consequently, the growing number of elderly people in India will be expected to contribute to a twofold rise in cancer incidence by the year 2040.^7^

The existing research on cancer in India has primarily focused on incidence rates, mortality rates, and the burden of cancer. Although some studies have explored the cancer burden among different states in India, they have been limited to specific years and specific types of cancer. Only one study conducted by Dhillon et al. in 2018^4^ used data from the Global Burden of Disease Study (GBD) 2016 to analyze patterns and trends of cancer among all age groups in the states of India. Additionally, the elderly population accounted for 9.15% of the total population in India in 2019,^23^ and they experienced the highest incidence rates of cancer.^5^ Therefore, the objective of this study is to examine the trends and patterns of the cancer burden among the elderly in different regions of India at a subnational level.

## 2 DATA AND METHODS

### 2.1 Data

Data were extracted from the GBD India Compare 2019.^24^ The estimates for this study were provided by the Institute for Health Metrics and Evaluation (IHME) in collaboration with the Indian Council of Medical Research (ICMR) and the Public Health Foundation of India (PHFI).^4^ Within the GBD India Compare, data were available for five aspects: cause, risk, etiology, impairment, and injuries by nature. Among these aspects, cancer falls under the causes of disease category (https://vizhub.healthdata.org/gbd‐compare/india).^24^ The causes of disease are categorized into four levels. Cancer (neoplasms) is located at level three, encompassing all causes of cancer. Further, level four provides more detailed information on specific causes of cancer, such as breast cancer, stomach cancer, and other causes of cancer.^25^ In this study data extracted at levels three and four of the causes of disease spanning from 1990 to 2019 to collect cancer information.

GBD India Compare^26^ provided data on the following age groups; 60–64 years, 65–69 years, and 70 years and above. By summing the cancer burden among these three age groups, we obtained the burden of cancer among the elderly. In addition, data on the prevalence of cancer cases and the accompanying DALY associated with cancer have been extracted to estimate the burden of cancer.

### 2.2 Methods

DALY is a standardized measure that consists of the loss of healthy life years from fatality (YLL) and the loss of healthy life years from nonfatality (YLD). The unit of measurement for DALY is years. One DALY represents the loss of 1 year of healthy life.^25^

(1) DALY=YLD+YLL

Murray (1994)^26^ and WHO (2020)^27^ have described the calculation of GBD data using the following formulas for YLD and YLL:

(2) $$\mathrm{YLD}=P_{x}\times \mathrm{DW}\times L_{x}$$

In Equation (2), *P*_x_ is the number of prevalence cases among age group x, DW is the disability weightage, and *L*_x_ is the average duration of illness among age group x in years. Disability weightage varies from zero to one. Zero disability weightage shows perfect health and one disability weightage shows death.

(3) $$\mathrm{YLL}=d_{x}\times e_{x}$$

In Equation (3), *d*_x_ is the number of fatality cases in different age groups, and *e*_x_ = expected standard life loss among different age groups. Expected standard life expectancy can be found by using the “abridged life table.”

To avoid an overestimation of the disease burden, adjustments should be made for the presence of comorbidity.^27^ It was assumed that the prevalence of one disease was independent of the other. The estimation of comorbidity was adjusted using Equation (4).

(4) $${\mathrm{YLD}}_{1+2}={\mathrm{YLD}}_{1}+{\mathrm{YLD}}_{2}-\left({\mathrm{YLD}}_{1}\times {\mathrm{YLD}}_{2}\right)$$

In Equation (4), YLD_1 + 2_ denotes the years of life lost due to the simultaneous presence of diseases 1 and 2. YLD_1_ represents the years of life lost due to morbidity from disease 1, while YLD_2_ represents the years of life lost from disease 2.

(5) $$\text{Total population}=\frac{\text{Total prevalence of cancer cases}}{\text{Prevalence of cancer cases}\;\mathrm{per}\;100,000\;\text{people}}\times 100,000$$

The GBD India Compare dataset provides information on the total prevalence of cancer cases and the prevalence of cancer cases per 100,000 people within the age groups of 60–64 years, 65–69 years, and 70 years and above. However, for this study, the analysis focused on the elderly. Therefore, it is necessary to sum up the total prevalence of cancer cases across these three age groups to obtain the total prevalence of cancer cases among the elderly. Additionally, when estimating the prevalence of cancer cases per 100,000 elderly, it is essential to have information on the total population of the elderly. Equation (5) was utilized in this study to obtain the total number of elderly.

(6) $$\text{Prevalence of cancer}\;\mathrm{per}\;100,000\;\text{people}=\frac{\text{Total prevalence of cancer cases}}{\text{Total population}}\times 100,000$$

(7) $$\text{DALY}\;\mathrm{per}\;100,000\;\text{people for cancer}=\frac{\text{Total DALY for cancer}}{\text{Total population}}\times 100,000$$

(8) $$\text{Annual}\%\text{change in prevalence of cancer cases}\;\mathrm{per}\;100,000\;\text{people}=\frac{\left(\frac{{\mathrm{PCC}}_{2019-{\mathrm{PCC}}_{1990}}}{{\mathrm{PCC}}_{1990}}\right)\times 100}{2019-1990}$$

In Equation (8), PCC_2019_ represents the prevalence of cancer cases per 100,000 people in the year 2019, and PCC_1990_ represents the prevalence of cancer cases in the year 1990. The numerator of this equation calculates the total percentage change in the prevalence of cancer cases per 100,000 people between the two time periods, while the denominator represents the number of years gap between the current year (2019) and the base year (1990). The annual percentage change in the prevalence of cancer cases per 100,000 people reflects the trends in the prevalence of cancer rate. A positive annual percentage change in the prevalence of cancer cases per 100,000 people indicates an increase in the prevalence of cancer rate per year and vice versa. The equation for the annual percentage change in DALY per 100,000 people due to cancer follows a similar structure as Equation (8).

## 3 RESULTS

**FIGURE 1:**
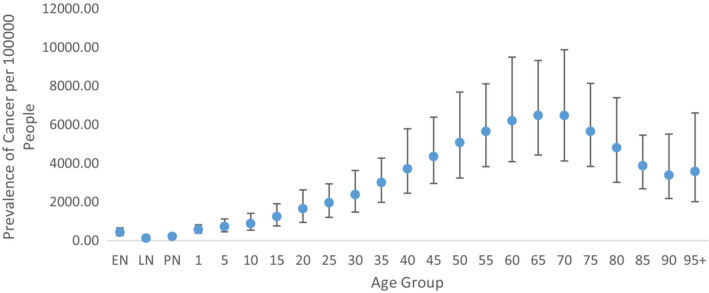
Age group–wise prevalence rate of cancer in India, 2019. *Source*: Authors' computation from the Global Burden of Disease Study 2019. EN = 0–6 days; LN = 7–27 days; PN = 28–364 days.

**FIGURE 2:**
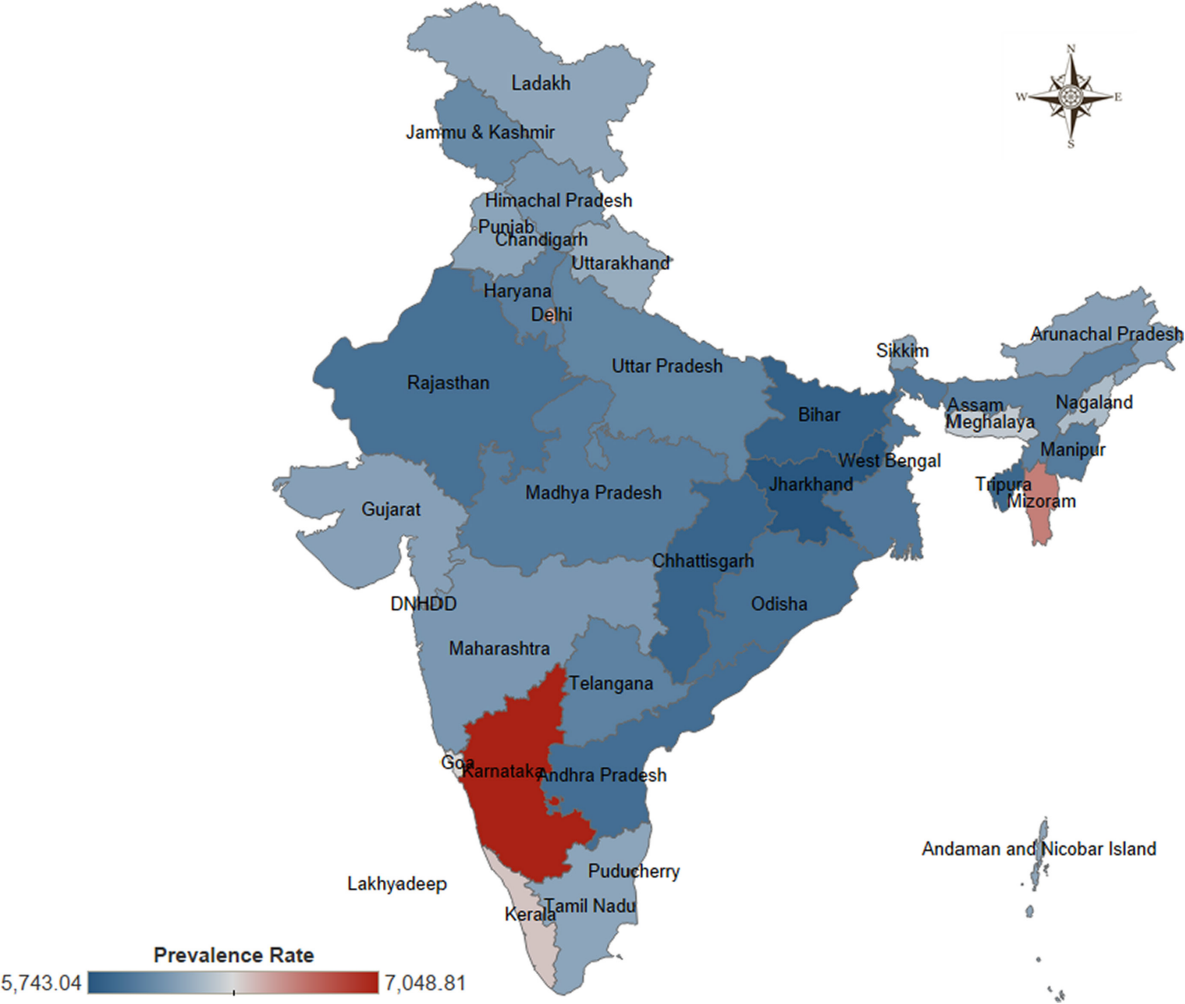
State‐wise prevalence rate of cancer among the elderly in India, 2019. *Source*: Authors' computation from the Global Burden of Disease Study 2019.

**FIGURE 3:**
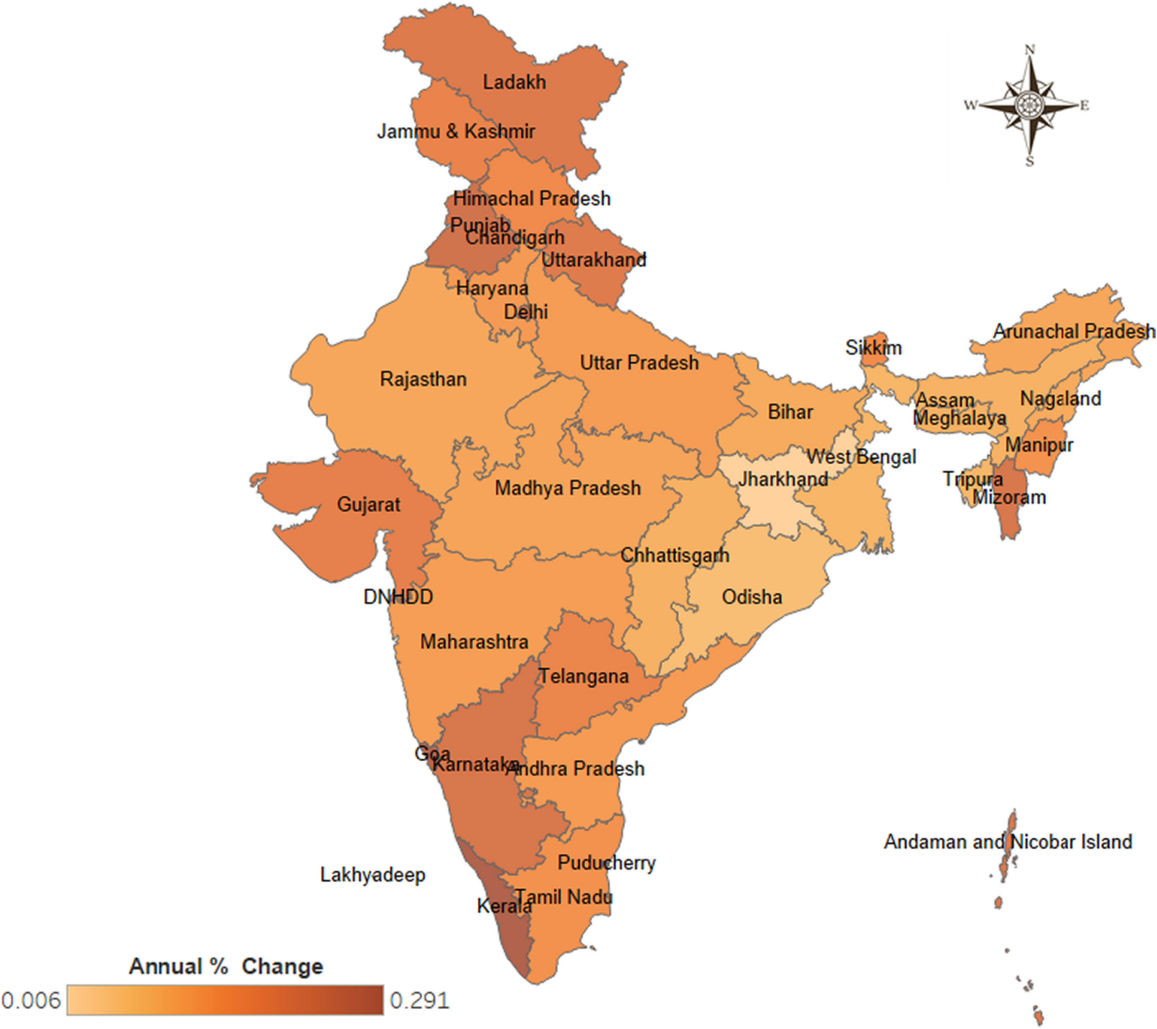
State‐wise annual percentage change in prevalence rate of cancer among the elderly in India, 1990–2019. *Source*: Authors' computation from the Global Burden of Disease Study 2019.

The prevalence of cancer per 100,000 people (prevalence rate) continuously increased with age up to 65–69 years and then declined. The top three age groups among whom the highest prevalence rate of cancer occurred were 60–64 years, 65–69 years, and 70–74 years, as presented in Figure 1. The prevalence rate of cancer varies from 131.776 [95% Uncertainty Interval (UI): 95.913–175.480] among the aged 7–27 days old child to 6484.075 (UI: 4430.365–9322.072) among the aged 65–69 years old people (Table S1). In addition, the statewise unequal spread of the prevalence rate for cancer in 2019 is reflected in Figure 2. The prevalence rate of cancer ranged from 7048.815 in Karnataka to 5743.040 in Jharkhand, as given in Table S2. The trends of the prevalence rate of cancer in terms of the annual percentage change are reflected in Figure 3. The highest annual percentage change in the cancer prevalence rate in Kerala (0.291) followed by Goa (0.280) and Panjab (0.227), with the lowest in Jharkhand (0.006) followed by Odisha (0.052) and Tripura (0.066). Among all states, the prevalence rate of cancer was raised as indicated by the positive annual percentage change in the prevalence rate of cancer (Table S2).

**FIGURE 4:**
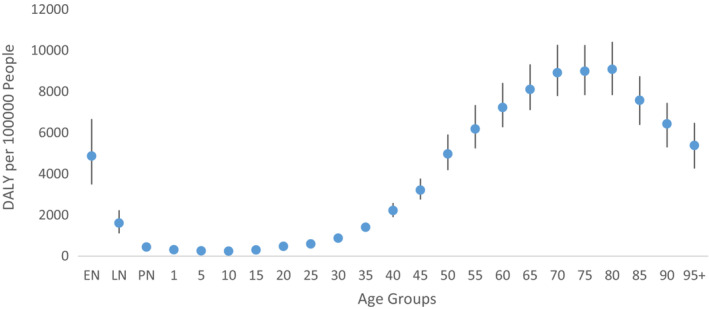
DALY rate of cancer across the age group in India, 2019. *Source*: Authors' computation from the Global Burden of Disease Study 2019. EN = 0–6 days; LN = 7–27 days; PN = 28–364 days.

**FIGURE 5:**
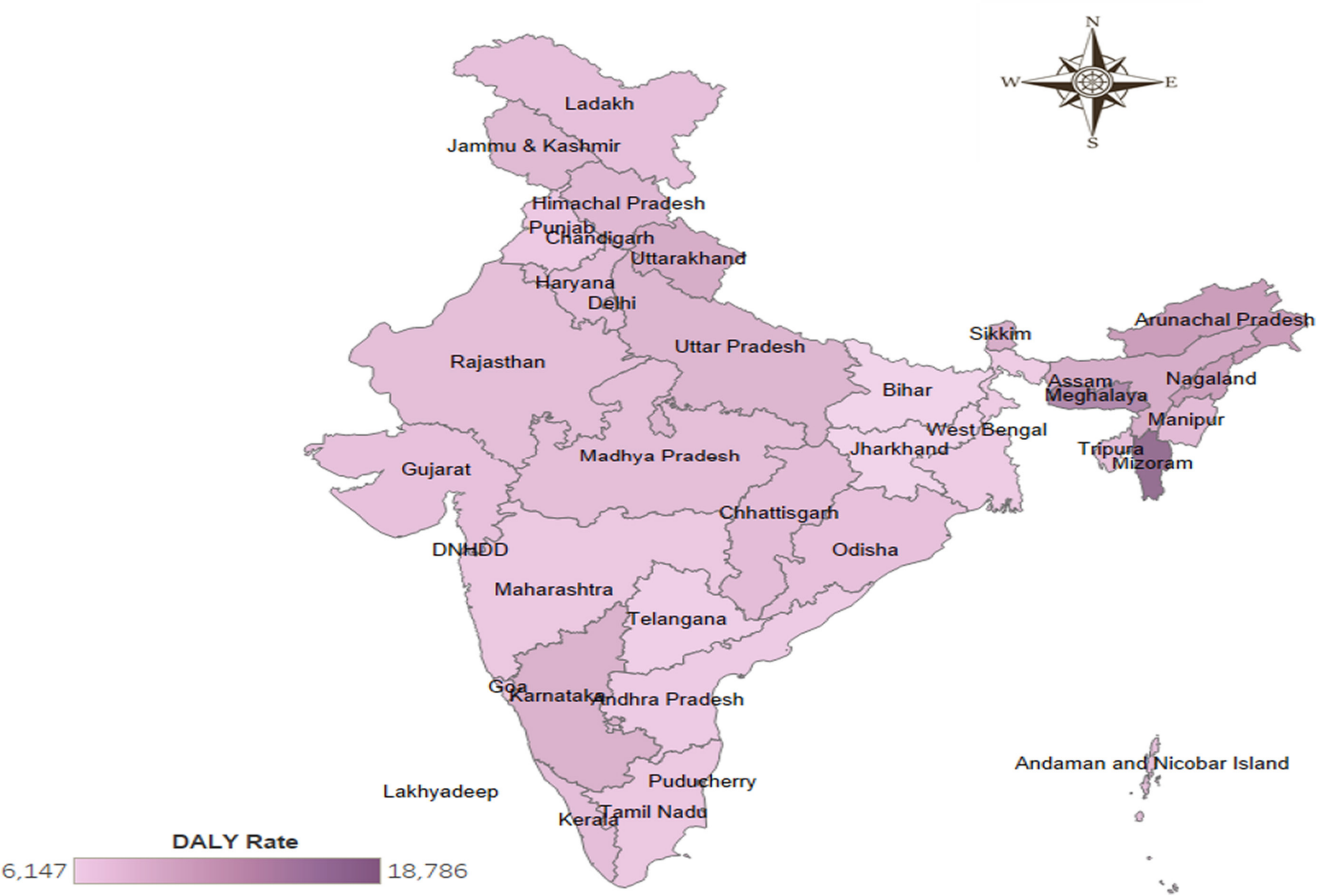
State‐wise DALY rate among the elderly for cancer in India, 2019. *Source*: Authors' computation from the Global Burden of Disease Study 2019.

The DALY per 100,000 people (DALY rate) due to cancer exhibited S‐shaped relationship across different age groups in 2019. This pattern was observed because the DALY rate consistently decreased with age, starting from 0 to 6 days (4869.186, UI: 3488.386–6672.559), reaching its lowest point among individuals aged 10–14 years (243.802, UI: 191.680–295.222). Subsequently, it progressively increased until it reached its peak among individuals aged 80–84 years (9087.825, UI: 7832.031–10421.190), after which it began to decline again, as presented in Figure 4 and Table S3. Figure 5 depicts the statewise burden of cancer among the elderly in 2019, represented by the DALY rate. The three states with the highest DALY rates were Mizoram (18786.476) followed by Meghalaya (15211.384) and Arunachal Pradesh (12322.608), while the three states with the lowest DALY rates were Bihar (6146.679) followed by Jharkhand (6210.706) and Telangana (6707.278). Furthermore, the DALY rate among the elderly in India in 2019 was 8112.283 (Table S4).

**FIGURE 6:**
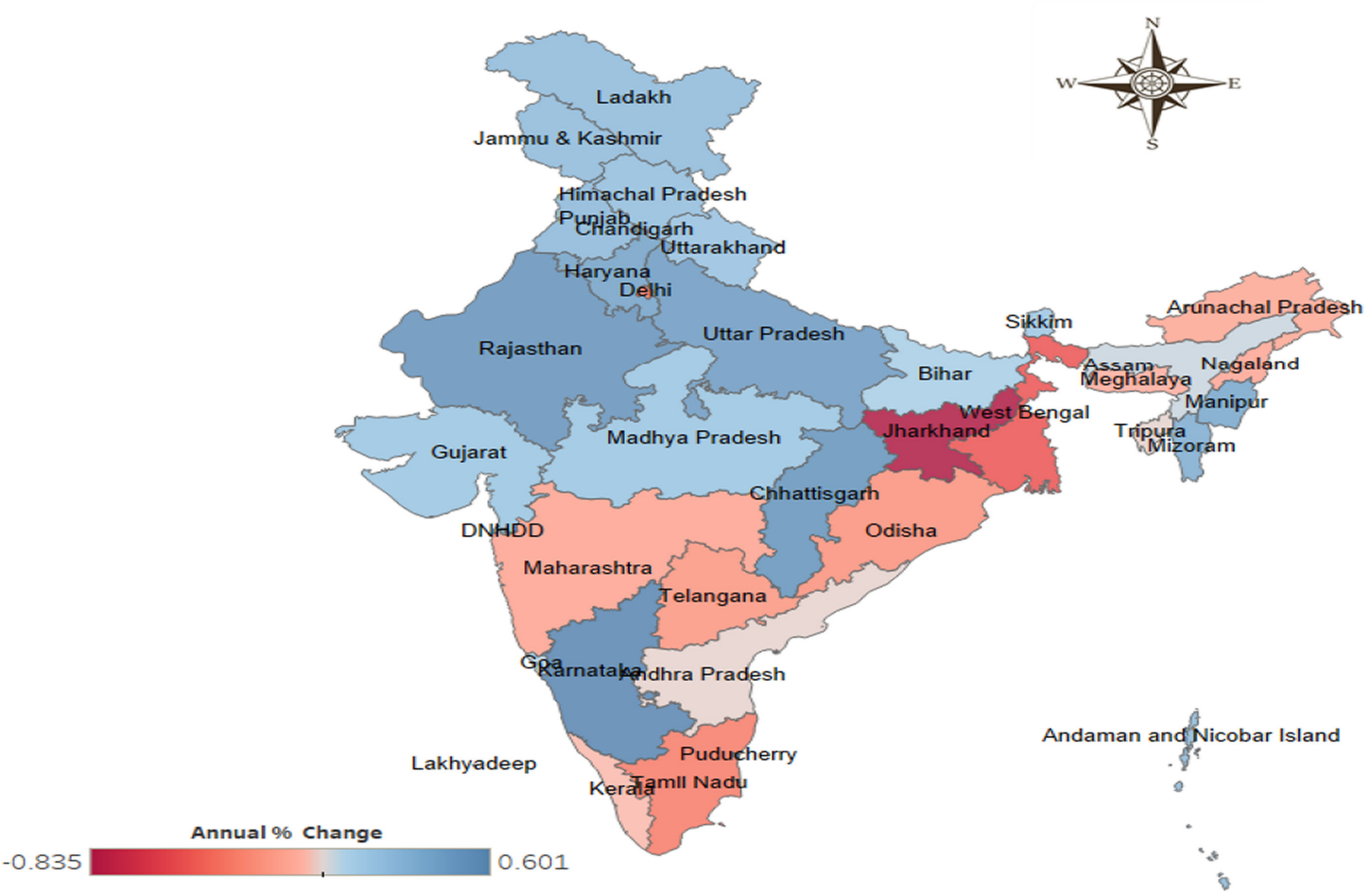
State‐wise annual percentage change in DALY rate among the elderly for cancer in India, 1990–2019. *Source*: Authors' computation from the Global Burden of Disease Study 2019.

Figure 6 presents the trends in the burden of cancer among the elderly from 1990 to 2019. During this period, 13 states and union territories experienced a decline in the DALY rate, indicating a negative annual percentage change. These states and union territories include Arunachal Pradesh (−0.127), Andhra Pradesh (−0.011), Delhi (−0.339), Kerala (−0.060), Maharashtra (−0.150), Meghalaya (−0.095), Nagaland (−0.132), Odisha (−0.236), Tamil Nadu (−0.328), Tripura (−0.009), West Bengal (−0.499), Jharkhand (−0.835), and Telangana (−0.212). On the other hand, the remaining states and union territories experienced a positive annual percentage change in the DALY rate among the elderly during this period (Table S4).

**FIGURE 7:**
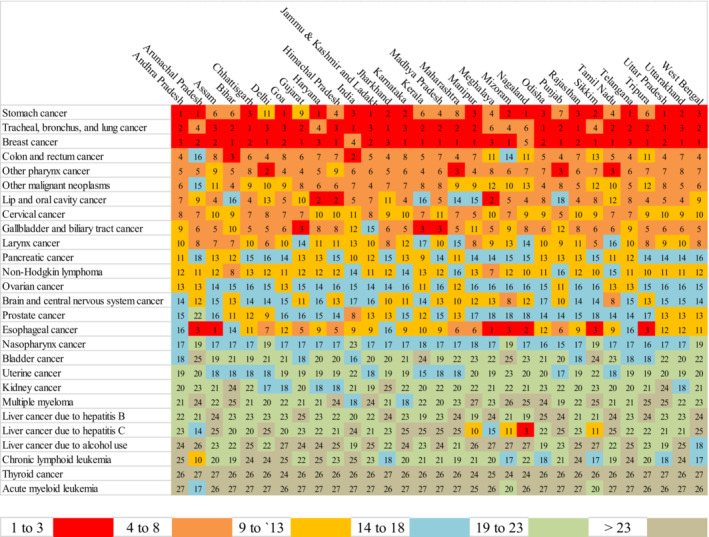
Heat map of cancers ranked by DALY rate among the elderly across the states of India, 2019 (in descending order). *Source*: Authors' computation from the Global Burden of Disease Study 2019. Rank of cancers in descending order.

**FIGURE 8:**
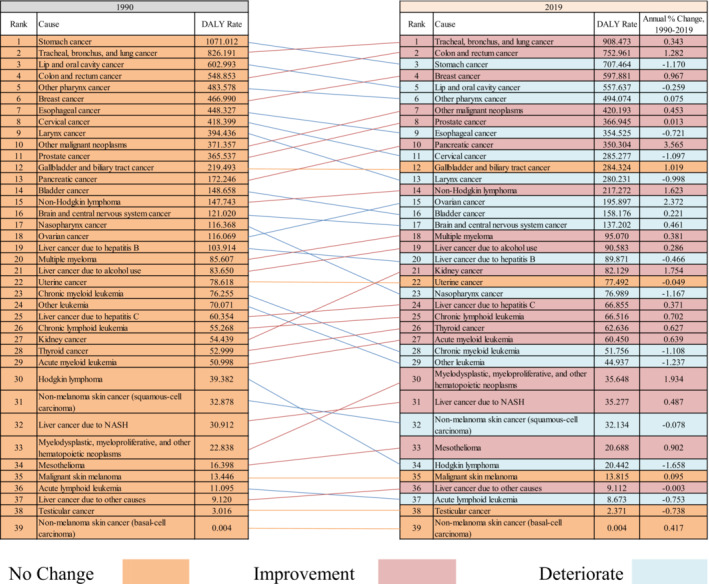
Change in the rank of types of cancers in terms of DALY rate among the elderly in India, 1990–2019. *Source*: Authors' computation from the Global Burden of Disease Study 2019. Change in rank for types of cancer among the elderly in 2019 compared to 1990.

The patterns of the burden of cancer among the elderly in 2019 were depicted in Figure 7 and was based on Table S5, which showed the ranking of cancers in terms of the DALY rate in descending order across states and in India. There is a disparity in the burden of cancers among states, as evidenced by the varying ranks of cancers from state to state. For example, the rank of tracheal, bronchus, and lung cancer varies from one to six across states. The top five cancers with higher DALY rates in India were tracheal, bronchus, and lung cancer (908.473), colon and rectum cancer (752.961), stomach cancer (707.464), breast cancer (597.881), and lip and oral cavity cancer (557.637) in 2019, as presented in Figure 8. In addition, over the year the change in the pattern of cancer based on the DALY rate. Out of the 39 types of cancers analyzed, the ranking improved for 18 cancers, deteriorated for 16 cancers, and remained unchanged for the remaining cancers. Furthermore, the highest annual percentage rise in the burden of pancreatic cancer, in terms of the DALY rate, was 3.565%, while the highest annual percentage fall was observed for Hodgkin lymphoma, with a decrease of 1.658% between 1990 and 2019.

## 4 DISCUSSION

In the elderly population, the higher prevalence rate of cancer compared to other age groups because of aging contributes to half of the overall increase in the global number of cancer patients between 2005 and 2015.^22^ Among the elderly, the age group of 65–69 years exhibits the highest prevalence rate of cancer, while the age group 80–84 years experiences the highest DALY rate. This discrepancy can be attributed to a combination of higher YLD and YLL rates in the age group 80–84 years compared to the age group 65–69 years.^24^ The prevalence rate of cancer among the elderly in India is more than twice as high (6078.146) as the average prevalence rate in the country (2571.890) in 2019. Similarly, the DALY rate among the elderly (8112.283) is more than four times the national average (1958.560) for the same year. Additionally, the prevalence rate demonstrates an annual percentage increase of 0.129, while the DALY rate shows an annual percentage increase of 0.053 between 1990 and 2019, contradicting previous studies.^22^ The reason for the higher rise in the prevalence rate over the 30 years in this study is due to its exclusive focus on the elderly population.

Stomach cancer, cervical cancer, larynx cancer, nasopharynx cancer, chronic myeloid leukemia, other leukemia, and Hodgkin lymphoma showed an annual decrease in DALY rate of more than 1%.^16^, ^17^, ^22^ Previous studies have indicated that the decline in stomach cancer can be attributed to the improved standard of living in India, which has led to increased consumption of fruits and vegetables known to have a positive effect against stomach cancer.^28^, ^29^ Additionally, other studies have suggested the transition of cancers from infectious to noninfectious as a country develops.^6^ Conversely, the top five cancers exhibiting the highest annual increase in DALY rate were pancreatic cancer, ovarian cancer, myelodysplastic, myeloproliferative, and other hematopoietic neoplasms, kidney cancer, and non‐Hodgkin lymphoma. However, these findings contradict the study by Dhillon et al.^16^ regarding ovarian cancer, which reported a decline in the annual percentage change of incidence between 1991 and 2005. The discrepancy can be attributed to variations in units of measurement and differences in the specific age groups under investigation. In addition, geographical variations in the states of India contributed to the unequal distribution of the cancer burden among states.^4^, ^12^

## 5 CONCLUSION

The prevalence rate of cancer among the elderly has been rising over the last 30 years across Indian states. The burden of cancer among the elderly is four times higher than that of the national average in India. The highest annual percentage rise in the burden of cancer is pancreatic cancer while the highest annual percentage fall in the burden is Hodgkin lymphoma. The burden of cancer among the elderly is the highest in Mizoram and lowest in Bihar.

This study helps to guide policymakers for the allocation of funds based on the prevalence rate and DALY rate. The cancer burden can be minimized by not only focusing on top‐rank cancers but also by focusing on cancers that have higher annual percentage increases in terms of the DALY rate. State‐specific government intervention should be taken to minimize the risk of cancer among the elderly. The present study has certain limitations that prevent it from addressing all questions regarding cancer in the elderly. Further detailed studies are required to answer specific questions regarding cancer among the elderly.

## AUTHOR CONTRIBUTIONS

CKS and HSR conceived the idea. CKS, SP, PKS, and HSR designed the study. CKS and SP collected the required data. CKS, SP, and US analyzed the data. HSR, US, and PKS interpreted the data. CKS and SP wrote the manuscript. All authors critically reviewed the manuscript and approved the final version submitted to Aging Medicine.

## FUNDING INFORMATION

This research did not receive any specific grant from funding agencies in the public, commercial, or not‐for‐profit sectors.

## CONFLICT OF INTEREST STATEMENT

The authors declare no conflicts of interest.

## Supporting information


Tables S1–S5.
