# Supplementary material for: Changing patterns of cancer burden among elderly across Indian states: Evidence from the global burden of disease study 1990–2019
Source: Aging Med (Milton). 2023 Aug 21;6(3):254–63. doi: 10.1002/agm2.12264 (PMC10498831; doi:10.1002/agm2.12264)
Supplement: Supplementary file 1 — Tables S1–S5. [file AGM2-6-254-s001.docx]

**Supplementary Tables**

**Table 1:** Age group-wise prevalence rate of cancer, 2019

| Age | Prevalence rate | 95% Uncertainty interval | | Rank of prevalence rate (Descending order) |
| --- | --- | --- | --- | --- |
|  |  | Lower bound | Upper bound |  |
| *0-6 days* | *434.752* | *262.847* | *664.818* | *21* |
| *7-27 days* | *131.776* | *95.913* | *175.480* | *23* |
| *28-364 days* | *219.495* | *142.816* | *315.352* | *22* |
| *1-4 years* | *574.917* | *382.843* | *817.748* | *20* |
| *5-9 years* | *729.036* | *455.967* | *1124.761* | *19* |
| *10-14 years* | *884.115* | *538.849* | *1416.032* | *18* |
| *15-19 years* | *1250.009* | *761.810* | *1909.860* | *17* |
| *20-24 years* | *1654.980* | *944.657* | *2626.792* | *16* |
| *25-29 years* | *1964.867* | *1207.836* | *2938.235* | *15* |
| *30-34 years* | *2386.045* | *1480.264* | *3630.612* | *14* |
| *35-39 years* | *3011.163* | *1983.059* | *4271.057* | *13* |
| *40-44 years* | *3716.952* | *2454.859* | *5791.277* | *10* |
| *45-49 years* | *4353.996* | *2962.385* | *6389.144* | *8* |
| *50-54 years* | *5078.131* | *3236.242* | *7691.914* | *6* |
| *55-59 years* | *5657.018* | *3825.825* | *8114.724* | *4* |
| *60-64 years* | *6206.887* | *4090.137* | *9496.400* | *3* |
| *65-69 years* | *6484.075* | *4430.365* | *9322.072* | *1* |
| *70-74 years* | *6477.383* | *4125.146* | *9875.916* | *2* |
| *75-79 years* | *5654.142* | *3839.806* | *8137.873* | *5* |
| *80-84 years* | *4806.453* | *3020.278* | *7399.248* | *7* |
| *85-89 years* | *3877.945* | *2684.935* | *5463.358* | *9* |
| *90-94 years* | *3395.377* | *2179.623* | *5512.515* | *12* |
| *95+ years* | *3580.415* | *2013.528* | *6611.530* | *11* |
| *All ages* | *2571.890* | *2110.940* | *3085.190* |  |

Source: Authors’ computation from the Global Burden of Disease Study 2019

**Table 2:** Prevalence rate and annual percentage change in prevalence rate of cancer among the elderly across state-wise in India, 1990-2019

| Locations | PR, 1990 | PR, 2019 | Rank of PR, 1990 | Rank of PR, 2019 | Total % Change in PR | Annual % Change in PR | Rank of annual % change in PR |
| --- | --- | --- | --- | --- | --- | --- | --- |
| Jammu & Kashmir and Ladakh | *5753.796* | *6034.099* | *23* | *17* | *4.872* | *0.168* | *10* |
| Arunachal Pradesh | *5974.998* | *6144.554* | *6* | *13* | *2.838* | *0.098* | *21* |
| Uttarakhand | *5869.070* | *6200.596* | *12* | *8* | *5.649* | *0.195* | *8* |
| Himachal Pradesh | *5832.326* | *6090.415* | *16* | *16* | *4.425* | *0.153* | *12* |
| Other Union Territories | *5838.807* | *6172.919* | *15* | *9* | *5.722* | *0.197* | *7* |
| Andhra Pradesh | *5685.173* | *5884.192* | *30* | *27* | *3.501* | *0.121* | *17* |
| Assam | *5858.793* | *5990.152* | *13* | *20* | *2.242* | *0.077* | *27* |
| Delhi | *6147.410* | *6541.862* | *4* | *3* | *6.417* | *0.221* | *4* |
| Goa | *5925.572* | *6406.206* | *9* | *5* | *8.111* | *0.280* | *2* |
| Haryana | *5775.406* | *5980.230* | *22* | *21* | *3.546* | *0.122* | *16* |
| Karnataka | *6645.191* | *7048.815* | *1* | *1* | *6.074* | *0.209* | *5* |
| Kerala | *5955.372* | *6457.236* | *7* | *4* | *8.427* | *0.291* | *1* |
| Maharashtra | *5906.298* | *6100.790* | *10* | *15* | *3.293* | *0.114* | *19* |
| Manipur | *5734.669* | *5957.576* | *26* | *23* | *3.887* | *0.134* | *15* |
| Meghalaya | *6188.457* | *6329.724* | *3* | *6* | *2.283* | *0.079* | *26* |
| Mizoram | *6298.531* | *6679.814* | *2* | *2* | *6.054* | *0.209* | *6* |
| Nagaland | *6110.226* | *6270.472* | *5* | *7* | *2.623* | *0.090* | *23* |
| Odisha | *5814.699* | *5901.837* | *18* | *25* | *1.499* | *0.052* | *30* |
| Punjab | *5778.572* | *6158.765* | *21* | *11* | *6.579* | *0.227* | *3* |
| Rajasthan | *5739.841* | *5900.560* | *24* | *26* | *2.800* | *0.097* | *22* |
| Sikkim | *5890.987* | *6142.525* | *11* | *14* | *4.270* | *0.147* | *13* |
| Tamil Nadu | *5930.703* | *6166.934* | *8* | *10* | *3.983* | *0.137* | *14* |
| Tripura | *5736.640* | *5846.034* | *25* | *28* | *1.907* | *0.066* | *29* |
| West Bengal | *5822.957* | *5940.652* | *17* | *24* | *2.021* | *0.070* | *28* |
| Bihar | *5659.360* | *5806.257* | *31* | *30* | *2.596* | *0.090* | *24* |
| Chhattisgarh | *5698.623* | *5828.890* | *29* | *29* | *2.286* | *0.079* | *25* |
| Jharkhand | *5733.863* | *5743.040* | *27* | *31* | *0.160* | *0.006* | *31* |
| Madhya Pradesh | *5788.956* | *5961.632* | *20* | *22* | *2.983* | *0.103* | *20* |
| Uttar Pradesh | *5809.477* | *6007.748* | *19* | *18* | *3.413* | *0.118* | *18* |
| Gujarat | *5841.124* | *6144.780* | *14* | *12* | *5.199* | *0.179* | *9* |
| Telangana | *5717.100* | *5990.500* | *28* | *19* | *4.782* | *0.165* | *11* |
| India | *5858.967* | *6078.146* |  |  | *3.741* | *0.129* |  |

Source: Authors’ computation from the Global Burden of Disease Study 2019

Note: PR = Prevalence rate

**Table 3:** Age group-wise DALY rate for cancer, 2019

| Age | DALY rate | 95% Uncertainty interval | | Rank of DALY rate (in descending order) |
| --- | --- | --- | --- | --- |
|  |  | Lower bound | Upper bound |  |
| 0-6 days | *4869.186* | *3488.386* | *6672.559* | *11* |
| 7-27 days | *1607.304* | *1105.451* | *2234.907* | *14* |
| 28-364 days | *443.048* | *337.516* | *580.343* | *19* |
| 1-4 years | *309.495* | *233.217* | *407.480* | *20* |
| 5-9 years | *254.172* | *195.147* | *318.609* | *22* |
| 10-14 years | *243.802* | *191.680* | *295.222* | *23* |
| 15-19 years | *301.197* | *254.758* | *346.440* | *21* |
| 20-24 years | *475.247* | *407.091* | *555.500* | *18* |
| 25-29 years | *591.721* | *503.408* | *693.873* | *17* |
| 30-34 years | *874.480* | *753.758* | *1022.650* | *16* |
| 35-39 years | *1403.994* | *1208.096* | *1638.412* | *15* |
| 40-44 years | *2217.715* | *1899.430* | *2582.376* | *13* |
| 45-49 years | *3212.832* | *2753.157* | *3773.393* | *12* |
| 50-54 years | *4968.243* | *4185.417* | *5918.330* | *10* |
| 55-59 years | *6185.534* | *5242.085* | *7347.638* | *8* |
| 60-64 years | *7231.897* | *6272.477* | *8428.046* | *6* |
| 65-69 years | *8110.867* | *7103.520* | *9331.896* | *4* |
| 70-74 years | *8921.231* | *7791.193* | *10275.509* | *3* |
| 75-79 years | *8992.369* | *7834.648* | *10268.053* | *2* |
| 80-84 years | *9087.825* | *7832.031* | *10421.190* | *1* |
| 85-89 years | *7580.646* | *6385.963* | *8750.537* | *5* |
| 90-94 years | *6433.300* | *5292.102* | *7454.886* | *7* |
| 95+ years | *5383.754* | *4261.054* | *6483.827* | *9* |
| All Ages | *1958.560* | *1708.330* | *2225.440* |  |

Source: Authors’ computation from the Global Burden of Disease Study 2019

**Table 4:** DALY rate and annual percentage change in DALY rate among elderly for cancer by state-wise in India, 1990-2019

| Locations | DALY rate, 1990 | DALY rate, 2019 | Rank of DALY rate , 1990 | Rank of DALY rate , 2019 | % Change in DALY rate | Annual % change in DALY rate | Rank of annual % change in DALY rate |
| --- | --- | --- | --- | --- | --- | --- | --- |
| Jammu & Kashmir and Ladakh | *8420.152* | *9021.537* | *14* | *12* | *7.142* | *0.246* | *10* |
| Arunachal Pradesh | *12793.139* | *12322.608* | *3* | *3* | *-3.678* | *-0.127* | *9* |
| Uttarakhand | *10005.918* | *10569.970* | *7* | *5* | *5.637* | *0.194* | *12* |
| Himachal Pradesh | *8603.374* | *9154.895* | *10* | *11* | *6.411* | *0.221* | *11* |
| Other Union Territories | *7690.989* | *8244.675* | *23* | *20* | *7.199* | *0.248* | *9* |
| Andhra Pradesh | *7151.179* | *7128.017* | *28* | *27* | *-0.324* | *-0.011* | *12* |
| Assam | *10375.121* | *10492.051* | *5* | *6* | *1.127* | *0.039* | *18* |
| Delhi | *10194.304* | *9191.508* | *6* | *10* | *-9.837* | *-0.339* | *3* |
| Goa | *7440.967* | *7587.527* | *26* | *23* | *1.970* | *0.068* | *17* |
| Haryana | *7555.885* | *8463.227* | *24* | *17* | *12.008* | *0.414* | *5* |
| Karnataka | *8526.496* | *10012.754* | *12* | *8* | *17.431* | *0.601* | *1* |
| Kerala | *8596.892* | *8448.121* | *11* | *18* | *-1.731* | *-0.060* | *11* |
| Maharashtra | *7835.813* | *7493.892* | *21* | *24* | *-4.364* | *-0.150* | *7* |
| Manipur | *7966.373* | *8832.508* | *20* | *13* | *10.872* | *0.375* | *6* |
| Meghalaya | *15640.000* | *15211.384* | *2* | *2* | *-2.741* | *-0.095* | *10* |
| Mizoram | *17110.277* | *18786.476* | *1* | *1* | *9.796* | *0.338* | *7* |
| Nagaland | *12671.092* | *12186.888* | *4* | *4* | *-3.821* | *-0.132* | *8* |
| Odisha | *8700.023* | *8105.207* | *9* | *22* | *-6.837* | *-0.236* | *5* |
| Punjab | *6764.568* | *7273.639* | *30* | *26* | *7.526* | *0.260* | *8* |
| Rajasthan | *7380.292* | *8483.953* | *27* | *16* | *14.954* | *0.516* | *2* |
| Sikkim | *9911.508* | *10348.086* | *8* | *7* | *4.405* | *0.152* | *15* |
| Tamil Nadu | *8203.740* | *7423.739* | *17* | *25* | *-9.508* | *-0.328* | *4* |
| Tripura | *8351.603* | *8330.389* | *15* | *19* | *-0.254* | *-0.009* | *13* |
| West Bengal | *8240.670* | *7049.241* | *16* | *28* | *-14.458* | *-0.499* | *2* |
| Bihar | *5958.966* | *6146.679* | *31* | *31* | *3.150* | *0.109* | *15* |
| Chhattisgarh | *7546.704* | *8661.716* | *25* | *14* | *14.775* | *0.509* | *3* |
| Jharkhand | *8195.579* | *6210.706* | *18* | *30* | *-24.219* | *-0.835* | *1* |
| Madhya Pradesh | *8153.309* | *8569.995* | *19* | *15* | *5.111* | *0.176* | *13* |
| Uttar Pradesh | *8431.146* | *9532.084* | *13* | *9* | *13.058* | *0.450* | *4* |
| Gujarat | *7756.894* | *8150.500* | *22* | *21* | *5.074* | *0.175* | *14* |
| Telangana | *7147.190* | *6707.278* | *29* | *29* | *-6.155* | *-0.212* | *6* |
| India | *7990.532* | *8112.283* |  |  | *1.524* | *0.053* |  |

Source: Authors’ computation from the Global Burden of Disease Study 2019

Note: Rank of annual % change in DALY Rate written in red color for positive annual percentage change of DALY rate and black color for negative annual percentage change of DALY rate. The positive annual percentage change of DALY rate is ranked in descending order and the negative annual percentage change of DALY rate is ranked in ascending order.

Table 5: State-wise DALY rate among the elderly by types of cancer, 2019

| Types of cancer | Locations | | | | | | | | | | | | | | | | | | | | | | | | | | | | | | |
| --- | --- | --- | --- | --- | --- | --- | --- | --- | --- | --- | --- | --- | --- | --- | --- | --- | --- | --- | --- | --- | --- | --- | --- | --- | --- | --- | --- | --- | --- | --- | --- |
|  | Andhra Pradesh | Arunachal Pradesh | Assam | Bihar | Chhattisgarh | Delhi | Goa | Gujarat | Haryana | Himachal Pradesh | Jammu & Kashmir and Ladakh | Jharkhand | Karnataka | Kerala | Madhya Pradesh | Maharashtra | Manipur | Meghalaya | Mizoram | Nagaland | Odisha | Punjab | Rajasthan | Sikkim | Tamil Nadu | Telangana | Tripura | Uttar Pradesh | Uttarakhand | West Bengal |  |
| Stomach cancer | *501.273* | *6943.690* | *332.173* | *173.714* | *361.502* | *190.041* | *1007.567* | *180.072* | *453.222* | *531.592* | *1049.154* | *312.040* | *480.619* | *229.024* | *291.310* | *188.942* | *696.297* | *2019.280* | *9392.578* | *4183.994* | *334.572* | *170.708* | *394.474* | *4774.608* | *239.218* | *453.714* | *485.025* | *480.371* | *709.873* | *238.066* |  |
| Colon and rectum cancer | *440.377* | *1799.203* | *400.784* | *322.879* | *456.827* | *382.276* | *886.705* | *395.098* | *393.198* | *579.379* | *537.465* | *321.459* | *465.192* | *427.720* | *395.631* | *427.915* | *797.055* | *1125.407* | *2602.196* | *1709.254* | *413.067* | *417.975* | *432.188* | *2480.542* | *387.357* | *432.395* | *675.804* | *428.059* | *581.390* | *291.702* |  |
| Tracheal, bronchus, and lung cancer | *407.740* | *3063.891* | *506.611* | *352.725* | *445.963* | *626.886* | *971.831* | *404.655* | *397.345* | *628.257* | *956.663* | *309.867* | *487.314* | *631.911* | *435.575* | *432.737* | *2189.057* | *2167.668* | *10474.077* | *1711.068* | *390.991* | *450.812* | *410.702* | *4982.604* | *390.030* | *414.548* | *1317.428* | *437.520* | *757.927* | *414.233* |  |
| Prostate cancer | *247.605* | *796.869* | *228.077* | *246.839* | *265.970* | *324.863* | *503.518* | *207.757* | *240.864* | *332.387* | *339.887* | *235.621* | *263.075* | *257.617* | *247.243* | *245.972* | *419.940* | *472.451* | *701.276* | *645.428* | *258.820* | *244.052* | *227.578* | *995.834* | *219.176* | *232.843* | *322.968* | *292.597* | *314.618* | *209.797* |  |
| Breast cancer | *243.137* | *1258.741* | *213.657* | *211.186* | *234.800* | *430.050* | *820.532* | *261.556* | *275.753* | *304.190* | *271.485* | *206.975* | *316.189* | *307.781* | *260.664* | *305.754* | *538.084* | *795.597* | *1630.621* | *1370.931* | *189.141* | *361.549* | *240.241* | *1517.371* | *249.695* | *213.343* | *428.785* | *225.711* | *291.801* | *162.375* |  |
| Other malignant neoplasms | *240.571* | *813.036* | *160.653* | *229.606* | *195.719* | *205.627* | *488.638* | *187.846* | *257.193* | *379.039* | *369.256* | *172.732* | *287.615* | *183.240* | *202.328* | *166.512* | *392.415* | *452.172* | *943.658* | *548.888* | *326.622* | *166.220* | *335.681* | *1004.556* | *156.086* | *231.443* | *283.880* | *196.335* | *432.974* | *173.925* |  |
| Other pharynx cancer | *195.541* | *999.898* | *368.883* | *54.743* | *311.340* | *154.711* | *639.820* | *130.969* | *452.819* | *602.867* | *261.441* | *114.720* | *441.258* | *85.045* | *282.639* | *81.202* | *204.182* | *2329.664* | *1793.840* | *1796.619* | *177.069* | *43.437* | *376.319* | *1488.727* | *96.116* | *184.816* | *570.201* | *276.857* | *473.701* | *130.725* |  |
| Pancreatic cancer | *185.372* | *1132.814* | *197.902* | *112.706* | *243.843* | *209.692* | *530.601* | *206.341* | *201.302* | *283.462* | *258.226* | *134.444* | *185.909* | *193.651* | *132.417* | *217.593* | *436.759* | *618.962* | *1132.842* | *819.755* | *164.669* | *238.432* | *156.589* | *1419.189* | *205.422* | *197.641* | *356.337* | *156.992* | *263.045* | *129.159* |  |
| Lip and oral cavity cancer | *173.151* | *1193.022* | *347.138* | *89.199* | *276.580* | *300.751* | *606.373* | *344.160* | *232.201* | *327.680* | *98.508* | *176.807* | *347.597* | *319.784* | *393.610* | *226.382* | *301.684* | *1574.259* | *1034.107* | *1055.612* | *245.669* | *101.906* | *183.150* | *1753.122* | *212.311* | *162.963* | *515.347* | *267.816* | *320.739* | *184.094* |  |
| Gallbladder and biliary tract cancer | *171.091* | *1012.810* | *311.686* | *126.464* | *191.594* | *265.373* | *346.819* | *77.215* | *151.440* | *199.064* | *189.408* | *149.217* | *153.688* | *70.253* | *174.126* | *78.682* | *408.871* | *619.790* | *829.183* | *520.250* | *153.743* | *144.928* | *152.374* | *1763.207* | *53.748* | *156.876* | *396.609* | *158.440* | *216.512* | *134.274* |  |
| Non-Hodgkin lymphoma | *155.965* | *581.611* | *121.384* | *68.938* | *107.917* | *116.247* | *269.798* | *84.212* | *96.271* | *144.175* | *125.076* | *81.108* | *131.888* | *171.279* | *97.293* | *121.229* | *208.411* | *345.608* | *675.467* | *359.360* | *94.199* | *92.581* | *105.480* | *721.223* | *107.949* | *96.026* | *205.966* | *88.623* | *140.742* | *67.708* |  |
| Larynx cancer | *114.386* | *844.066* | *154.527* | *120.071* | *154.831* | *169.956* | *326.105* | *88.703* | *108.012* | *175.531* | *167.549* | *91.259* | *115.280* | *92.815* | *125.554* | *78.450* | *211.295* | *802.495* | *900.906* | *729.529* | *116.231* | *61.833* | *116.795* | *1284.611* | *63.687* | *115.755* | *323.273* | *154.439* | *213.980* | *100.886* |  |
| Ovarian cancer | *94.851* | *821.121* | *97.730* | *57.327* | *90.663* | *122.638* | *270.612* | *76.258* | *90.728* | *114.749* | *103.196* | *84.132* | *104.738* | *114.988* | *88.912* | *96.937* | *186.462* | *261.511* | *473.805* | *337.512* | *81.495* | *103.599* | *70.299* | *720.218* | *83.379* | *88.434* | *143.950* | *65.817* | *112.009* | *68.188* |  |
| Cervical cancer | *92.016* | *836.596* | *97.688* | *67.396* | *130.059* | *142.944* | *204.090* | *98.149* | *70.664* | *157.750* | *78.222* | *128.754* | *173.276* | *91.801* | *119.200* | *143.694* | *254.046* | *412.783* | *1110.545* | *634.453* | *58.051* | *103.644* | *88.707* | *827.362* | *172.944* | *80.162* | *230.457* | *79.388* | *128.970* | *69.101* |  |
| Bladder cancer | *90.346* | *217.747* | *86.126* | *79.932* | *174.037* | *206.243* | *185.942* | *75.547* | *88.391* | *150.450* | *121.714* | *84.265* | *105.879* | *104.800* | *92.509* | *89.944* | *160.827* | *166.896* | *396.574* | *194.209* | *91.791* | *78.328* | *80.995* | *545.774* | *69.020* | *83.339* | *87.595* | *101.920* | *141.314* | *83.374* |  |
| Esophageal cancer | *73.029* | *2263.877* | *583.591* | *60.512* | *178.292* | *218.552* | *292.188* | *225.343* | *205.082* | *396.881* | *227.224* | *57.178* | *250.140* | *132.309* | *190.490* | *218.868* | *429.908* | *5024.984* | *3973.027* | *2388.257* | *112.253* | *231.479* | *163.100* | *3580.205* | *164.550* | *70.218* | *583.548* | *109.059* | *199.986* | *104.142* |  |
| Brain and central nervous system cancer | *59.090* | *388.654* | *53.659* | *48.253* | *66.434* | *71.497* | *168.389* | *43.675* | *58.505* | *87.534* | *75.211* | *54.161* | *62.221* | *59.276* | *52.524* | *54.779* | *138.376* | *205.123* | *345.596* | *271.068* | *59.165* | *62.119* | *53.544* | *448.807* | *49.724* | *57.240* | *110.600* | *53.453* | *82.252* | *42.108* |  |
| Chronic lymphoid leukemia | *50.446* | *147.010* | *35.205* | *31.102* | *46.546* | *41.854* | *87.374* | *38.446* | *40.100* | *51.016* | *49.973* | *38.095* | *45.036* | *31.483* | *38.114* | *32.705* | *70.130* | *93.947* | *164.569* | *122.367* | *36.656* | *40.851* | *39.323* | *281.583* | *25.496* | *43.124* | *70.332* | *37.436* | *55.377* | *28.635* |  |
| Multiple myeloma | *43.414* | *368.531* | *44.193* | *36.208* | *50.321* | *79.383* | *104.860* | *38.432* | *41.266* | *60.247* | *57.538* | *40.835* | *51.100* | *89.913* | *44.581* | *42.645* | *88.369* | *114.803* | *188.757* | *153.741* | *40.863* | *55.625* | *37.564* | *329.137* | *36.570* | *41.285* | *70.299* | *41.745* | *56.080* | *35.218* |  |
| Kidney cancer | *39.713* | *195.577* | *32.678* | *24.712* | *40.244* | *82.303* | *158.584* | *36.247* | *41.930* | *66.881* | *56.193* | *27.622* | *44.474* | *52.471* | *31.060* | *41.509* | *80.270* | *103.406* | *188.998* | *151.788* | *33.196* | *37.011* | *33.305* | *282.933* | *32.029* | *37.796* | *58.107* | *32.571* | *62.588* | *33.573* |  |
| Uterine cancer | *39.622* | *156.264* | *30.060* | *24.090* | *42.582* | *51.943* | *84.337* | *29.904* | *36.355* | *41.013* | *40.259* | *34.791* | *56.839* | *42.385* | *34.681* | *32.099* | *27.452* | *68.453* | *141.516* | *82.459* | *32.577* | *42.328* | *31.633* | *191.192* | *30.104* | *34.407* | *27.390* | *30.691* | *48.242* | *27.854* |  |
| Thyroid cancer | *36.291* | *319.700* | *24.857* | *26.256* | *40.207* | *33.695* | *80.001* | *18.181* | *32.280* | *44.076* | *41.736* | *31.317* | *39.731* | *55.841* | *29.481* | *28.764* | *109.784* | *65.382* | *225.670* | *166.006* | *32.477* | *14.542* | *31.789* | *380.307* | *22.799* | *28.504* | *32.293* | *33.074* | *45.437* | *21.591* |  |
| Nasopharynx cancer | *32.420* | *816.960* | *20.715* | *35.106* | *43.203* | *15.530* | *103.424* | *24.969* | *32.446* | *48.571* | *46.058* | *32.699* | *31.728* | *12.483* | *26.251* | *15.650* | *305.381* | *273.469* | *914.407* | *2239.381* | *33.861* | *6.457* | *31.407* | *1047.797* | *16.454* | *28.762* | *49.015* | *39.478* | *53.919* | *20.728* |  |
| Liver cancer due to hepatitis C | *31.951* | *137.585* | *28.163* | *26.779* | *11.221* | *33.740* | *13.539* | *22.379* | *30.089* | *32.799* | *31.436* | *34.444* | *39.436* | *33.272* | *29.270* | *38.020* | *34.369* | *41.949* | *120.443* | *41.939* | *41.352* | *30.033* | *25.344* | *82.955* | *27.650* | *20.403* | *34.415* | *49.671* | *33.876* | *51.134* |  |
| Liver cancer due to alcohol use | *31.556* | *855.154* | *35.139* | *36.111* | *17.151* | *24.733* | *34.401* | *27.495* | *23.883* | *43.526* | *40.623* | *53.465* | *49.739* | *47.529* | *31.867* | *42.643* | *83.122* | *105.191* | *448.926* | *128.530* | *53.577* | *39.406* | *26.088* | *552.037* | *35.814* | *22.102* | *65.730* | *51.753* | *39.251* | *61.482* |  |
| Acute myeloid leukemia | *1.526* | *121.363* | *2.769* | *1.142* | *4.748* | *8.661* | *58.338* | *1.794* | *4.287* | *13.409* | *9.608* | *3.113* | *1.807* | *3.057* | *1.427* | *0.915* | *37.191* | *52.990* | *174.212* | *89.760* | *2.468* | *3.246* | *1.784* | *118.645* | *1.172* | *2.584* | *19.520* | *0.634* | *11.503* | *0.880* |  |
| Liver cancer due to hepatitis B | *0.779* | *599.350* | *3.213* | *0.735* | *1.554* | *4.164* | *16.175* | *1.071* | *1.928* | *7.548* | *6.151* | *3.255* | *1.466* | *1.853* | *0.916* | *0.624* | *35.677* | *51.081* | *326.295* | *67.731* | *2.433* | *1.682* | *1.072* | *406.567* | *0.649* | *1.023* | *21.476* | *0.671* | *6.397* | *1.392* |  |

Source: Authors’ computation from the Global Burden of Disease Study 2019
